# Deliberate and Self-Conscious Adaptation of Eye-Contact by Autistic Adults

**DOI:** 10.1007/s10803-024-06296-4

**Published:** 2024-05-10

**Authors:** Alison Garvey, Christian Ryan, Mike Murphy

**Affiliations:** School of Applied Psychology, University College Cork, Distillery House, North Mall, Cork, T23 TK30 Ireland

**Keywords:** Autism Spectrum Disorder, Eye-contact, Eye gaze, Qualitative, Interpretative Phenomenological Analysis

## Abstract

Eye gaze is widely recognised as an important element in managing social interactions, receiving information from others and communicating information about ourselves. Atypical eye gaze is one of the characteristic hallmarks of autism. Experimental research has contributed significantly to our knowledge of eye-gaze in autism, however, there is a lack of research exploring how autistic individuals describe and understand the phenomenological experience of eye-gaze and eye-contact. The current study explores the subjective experience of deliberate and self-conscious adaptation of eye-contact by autistic adults. Nine autistic adults were interviewed individually about their experiences of eye-contact. Audio recordings were transcribed, and analysed using Interpretative Phenomenological Analysis (IPA). Themes included gaining awareness of eye contact differences through feedback from others, the interaction of motivation to make eye-contact with a sense of autistic identity, difficulties listening when making eye contact, the importance of familiarity of conversational partners, and strategies to make eye-contact. This study makes an important and novel contribution to understanding the experience of eye-contact differences from the perspective of autistic adults themselves, highlighting the phenomenology of eye-contact as potentially aversive and the social pressure to engage in it, and exploring factors influencing effective eye-contact, with implications for psychological intervention.

## Introduction

Eye gaze is widely recognised as a powerful tool for social interaction that has multiple functions in the modulation of social processes (Hietanen, 2018). Eye gaze serves a dual purpose, as we can both receive information from others, and use our gaze to communicate information (Cañigueral & Hamilton, 2019). For example, eye gaze allows us to detect what other people are looking at (Cañigueral & Hamilton, 2019), infer how others think and feel (Baron-Cohen et al., 1997; Cañigueral & Hamilton, 2019), and strategically cue another person’s attention (Cañigueral & Hamilton, 2019; Kuhn et al., 2009). Furthermore, eye gaze regulates and organises social interactions. For example, during conversation, the speaker ends their turn by shifting their gaze to the person listening, who in turn begins to speak with an averted gaze (Ho et al., 2015; Laskowitz et al., 2022). Thus, eye gaze is a significant factor in facilitating social interaction, and signalling turn-taking in conversations (Laskowitz et al., 2022).

This wide range of potential eye gaze behaviours includes a specific subtype, eye-contact, which is defined as two people looking simultaneously into each other’s eyes (Kleinke, 1986), and is associated with elevated physiological arousal (Senju & Johnson, 2009), and more specifically with affective arousal (Hietanen, 2018). In typical development, direct mutual eye-contact is often experienced positively (Hietanen, 2018). Indeed, a preference for eye-contact has been demonstrated through research that has found that typically developing adults (Senju & Hasegawa, 2005) and infants (Farroni et al., 2002) shift their attention preferentially and reflexively towards others’ direct gaze to establish mutual eye-contact. Conversely, one of the characteristic hallmarks of Autism Spectrum Disorder (ASD) includes an atypical response to eye-contact (Madipakkam et al., 2017). In his seminal article on ‘infantile autism’, Kanner (1943) described children who presented with ‘markedly unusual’ eye-contact and eye-gaze, including atypical eye-contact patterns and looking at socially irrelevant stimuli, and subsequent research has supported these observations (Frazier et al., 2017). Since that time, “abnormalities in eye contact” have been listed in the diagnostic criteria of ASD (DSM-5, American Psychiatric Association, 2013) and “unusual eye contact” in instruments such as the Autism Diagnostic Observation Schedule (Lord et al., 2000).

Memoirs by autistic adults, such as Robison (2007), have described how alien eye-contact may feel to autistic people ‘[we] are just not comfortable doing it. In fact, I don’t really understand why it’s considered normal to stare at someone’s eyeballs.’ Eye-tracking research has also contributed to our knowledge of atypical attention processes in ASD, and has provided evidence to suggest that differences in social attention are key features. However, it is important to note two key assumptions implicit in eye tracking research. Firstly, eye fixations are assumed to represent visual attention – that is, when a person fixates on a particular point in the visual field, it is assumed that they are currently attending to that point or object of interest. Secondly, it is assumed that visual attention (rather than other senses) is the most salient measure of attentional processes (Holmqvist et al., 2011). In contrast to typically developing comparison groups, social attention differences have been observed in autistic participants across studies, ranging from decreased fixation to others’ eyes and social stimuli in infancy (Chawarska et al., 2013; Jones & Klin, 2013), to less attentional bias for faces in autistic adults (Moore et al., 2012). Furthermore, recent meta-analyses of eye-tracking studies have found mixed results regarding social attention in ASD, but identified overall atypical gaze patterns in autistic individuals, characterised by reduced time attending to social stimuli, and difficulties selecting socially relevant information for attention (Chita-Tegmark, 2016; Frazier et al., 2017).

Despite evidence suggesting that autistic people exhibit atypical eye-contact, the underlying cause of this difference remains unclear. Four models make different predictions in an attempt to explain why autistic people might demonstrate reduced eye-contact. For example, the hyperarousal model proposes that eye-contact during mutual gaze directly activates brain arousal systems and an emotional response. Thus, from this perspective, autistic individuals purposefully avoid looking at others’ eyes, because the eyes are perceived as having negative valence (Moriuchi et al., 2017). Conversely, the hypoarousal model postulates that autistic people demonstrate reduced eye-contact as the amygdala fails to prioritise social information in the environment, and consequently, autistic individuals do not preferentially attend to social stimuli such as faces and eyes. Therefore, based on this model, social information has less intrinsic reward (Trevisan et al., 2017). Baron-Cohen’s (1994) ‘mindblindness’ model proposes that during typical development aspects of gaze perception are influenced by innate modules, including an ‘eye direction detector’, ‘shared attention mechanism’, and the ‘theory of mind mechanism’. Therefore, the lack of optimal functioning from one or more of these modules may reduce the degree to which autistic people attend to other peoples’ eyes to determine their intentions and mental states (Trevisan et al., 2017). Finally, the fast-track modulator model proposes a fast subcortical face detection pathway that modulates neural processing in cortical areas (Akechi et al., 2014; Senju & Johnson, 2009). From this perspective, it has been suggested that autistic people are impaired in the fast sub-cortical processing of information from other peoples’ eyes, which may subsequently alter the development of social cognition and ‘social brain’ networks, which are both related to difficulties associated with ASD (Akechi et al., 2014; Falck-Ytter et al., 2013; Senju & Johnson, 2009).

Although eye-tracking research has contributed important information about eye-contact in ASD, experimental research must focus on behaviour during tightly controlled, and primarily contrived, scenarios, giving us limited insight into real-world social attention. To date, we could find only one qualitative study of the subjective experience of eye-contact in autism. This was an analysis of YouTube and WrongPlanet videos of people with self-declared ASD describing their experience of eye-contact (Trevisan et al., 2017). However, there has been no qualitative interview studies exploring autistic peoples’ experience of eye-contact. Atypical eye-contact can lead to significant barriers and challenges for autistic people when attempting to regulate real-world social interactions (Trevisan et al., 2017). By including the voice of autistic people in research, we can develop an understanding of how autistic people experience eye-contact, and how this may inform psychological supports and intervention and potential social and environmental adaptations.

### The Present Study

In order to better understand the experience of eye-contact the current study aims to explore the subjective experience of deliberate and self-conscious adaptation of eye-contact by autistic individuals. In order to gain a richer understanding of participants’ experiences of eye-contact, factors such as strategies to make eye-contact and experience of eye-contact during social interactions will also be explored.

## Method

A member of the ASD community was consulted as an expert by experience to discuss the nature of the research and design of the data collection. This was done as a three-way online meeting with two of the authors, where we examined the wording of the interview schedule, the information sheet, and the use of video recording. We also decided on individual interviews rather than focus groups.

### Methodological Approach and Study Design

A qualitative design using Interpretative Phenomenological Analysis (IPA; Smith et al., 2021) and semi-structured interviews was the chosen method of data collection and analysis. IPA focuses on specific phenomena allowing for the close analysis of the details of a particular aspect of an individual’s experience in the world (Pietkiewicz & Smith, 2014; Smith, 2004; Smith et al., 2021), such as eye-contact. IPA is rooted in three philosophical traditions; phenomenology, hermeneutics, and idiography. It is a phenomenological approach as it is interested in the participants’ experience of living through or with a specific phenomenon. Research based in IPA adopts a double hermeneutic process, in which there is a dual process of interpretation; firstly at the level of the participant who is making meaning of their own lived experience, and secondly at the level of the researcher who is making sense of the participants’ sense-making (Smith et al., 2021). Finally, a core feature of IPA is its idiographic emphasis which aims to generate rich and detailed descriptions of how each individual participant makes meaning of the phenomena under question (Cooper et al., 2022). IPA has been deemed an effective qualitative approach in autism research, due to its reflexive engagement, and attempt to equalise the power between autistic participants and non-autistic researchers (Howard et al., 2019).

### Participants

Inclusion criteria specified that participants must be eighteen years of age or over and have no intellectual disability (ID). In line with theoretical underpinnings of IPA, a small purposeful sample of participants was recruited in order to identify a specific group for whom the research focus had relevance and personal significance (Pietkiewicz & Smith, 2014).

Nine participants with an ASD diagnosis were interviewed. Participants’ diagnoses were verified while accessing support services in which recruitment took place, as participants were required to have a clinical diagnosis of ASD from a psychologist or psychiatrist to access services. Participants were also asked to confirm their ASD diagnosis in the consent form. Participants ranged in age from 20 to 56 years, and seven were male, which is relatively reflective of the gender ratio typically observed in those with an ASD diagnosis (Elsabbagh et al., 2012). Demographic information for participants is presented in Table 1.

**Table 1:** *Demographic information*

Pseudonym	Sex (M/F)	Age (in years)
Maria	F	20
John	M	56
Sam	M	28
Kevin	M	44
Ryan	M	27
Jason	M	20
Lauren	F	21
Shane	M	20
Jack	M	28

### Procedure

Ethical approval was granted by the appropriate institutional ethical review panel at the local university. Participants were recruited from an ASD support service in Ireland. Participants were invited to keep a diary of their experience of eye-contact for one week before the scheduled interview, to enrich the recall of the phenomenological aspects of eye-contact experienced immediately prior to the interviews. Recruitment took place during the COVID-19 pandemic, so data was collected online using a live video interview. Participants were provided with an electronic version of the information sheet prior to attending the interview, outlining the purpose of the research, as well as informing them of their rights regarding participation. Participants needed a reliable internet connection, a smartphone or laptop, and a private space in order to participate. Most participants reported that they had experience of using online video platforms in the context of COVID-19 and all participants had the necessary access to Wi-Fi to participate. Participants were informed, prior to participation, that the interview would be video-recorded, and were given the opportunity to ask questions or take a break at any time during the interview.

The interview schedule covered the following topics: eye-contact growing up and in the family, experiences of social skills training, strategies to make eye-contact, and the experience of eye-contact itself. Adaptations for conducting semi-structured interviews with autistic participants included piloting the interview schedule (Maloret & Scott, 2018; Tierney et al., 2016), and providing participants with an outline of topics to be discussed prior to the interview (Griffith et al., 2012; Huws & Jones, 2015; MacLeod et al., 2018; Petalas et al., 2015).

Information on how to access the meeting was included in the information sheet, and participants received an email with a link to the meeting prior to attending. The interview was video recorded using online software and transcribed by the first author. The mean length of the interviews was 51 min, with a range of 37–66 min.

### Data Analysis

Data analysis followed the seven sequential steps recommended by Smith et al. (2021). This involved reading and re-reading the transcripts, and then making ‘exploratory notes’ to capture descriptive, linguistic and conceptual aspects of the data. Experiential statements were then generated by analysing the exploratory notes of discrete chunks of transcript. The next step involved searching for connections across experiential statements, and producing a structure of the most interesting and important aspects of the participants’ account. Each cluster of experiential statements were then labelled as ‘personal experiential themes’ (PETs), and consolidated and organised in a table. This systematic process of idiographic, individual analysis was continued for each participant. Finally, patterns of convergence and divergence were searched for across PETs to develop a set of ‘Group Experiential Themes’ (GETs), to highlight the shared and unique characteristics of the experience of eye-contact across all participants (Smith et al., 2021).

### Validity and Quality Assurance

Nizza et al.’s (2021) guidance for researchers to produce excellence in IPA research was applied to ensure credibility and trustworthiness of the analysis. Credibility of analysis was also ensured through a coding audit, which was completed by the second author in order to ensure that the exploratory notes, experiential statements and themes were grounded in the transcripts and the participants’ reported experiences and intended meaning (Smith et al., 2021). Finally, the first author attended IPA meetings with the second author to discuss the methodology, and critically evaluate the analysis of a sample of transcripts. The first author kept a reflexive diary throughout the research process, and aimed to stay close to the meaning-making of each individual in order to offer insights into the participants’ experience, while remaining aware of her identity as a non-autistic researcher. The research team engaged in reflexive bracketing, that is, attempting to note preconceptions and assumptions, but also staying aware of moments of surprise in the research, when previously undiscovered preconceptions emerge from the iterative process of being confronted with a differing view from a participant. This is a key part of the working through the hermeneutic circle in IPA during which both the participant and the researcher are engaged in meaning making (Smith et al., 2021).

## Results

**Fig. 1:**
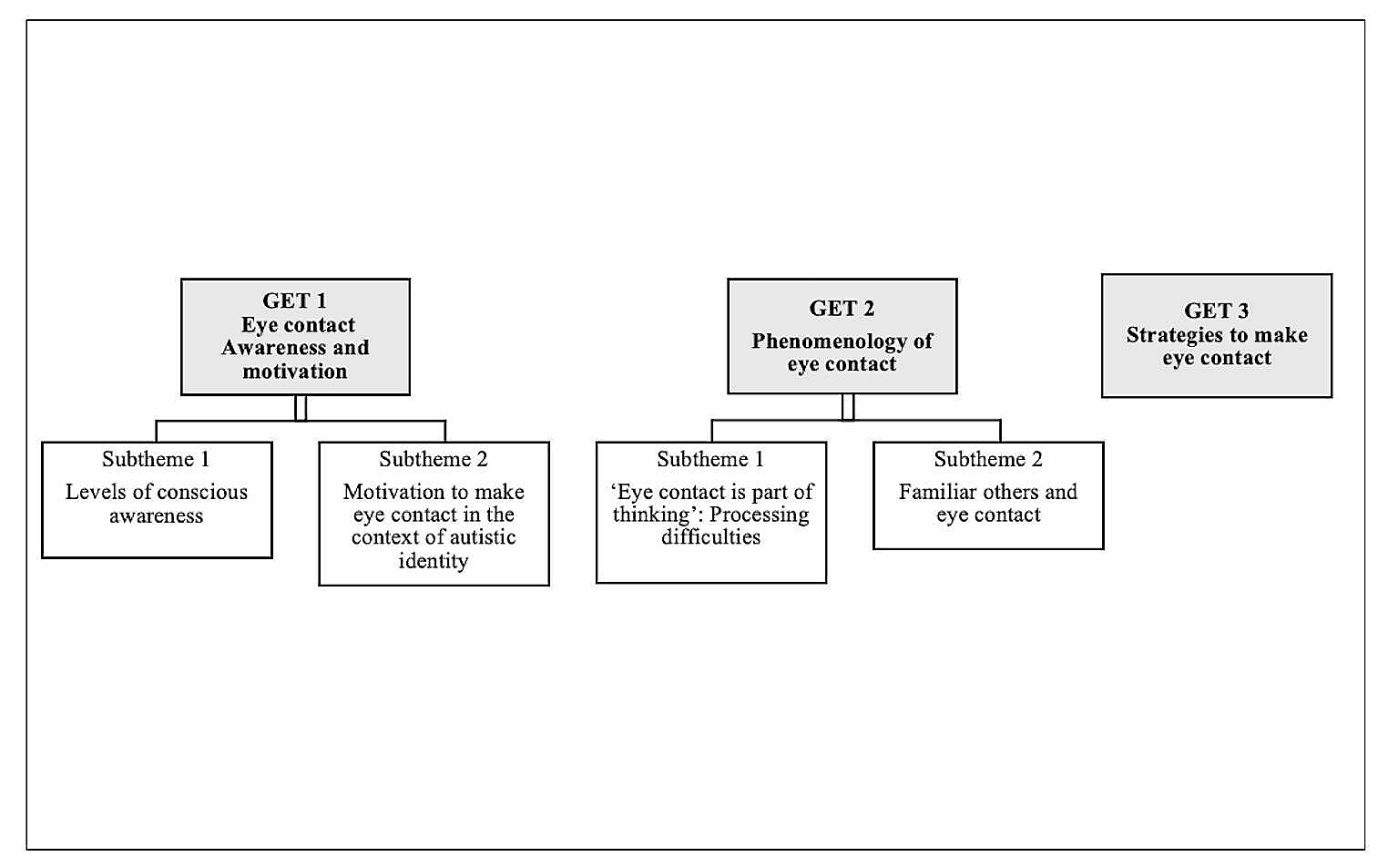
Group experiential themes and subthemes

Analysis of the data produced three interrelated group experiential themes (See Fig. 1).

### GET 1: Eye-contact: Awareness and Motivation

This GET reflects participants’ levels of conscious awareness of the atypicality of their eye-contact, as well as their motivations to make eye-contact in the context of their autistic identity.

#### Subtheme 1: Levels of Conscious Awareness

For most participants, atypical eye-contact was experienced outside of conscious awareness, and entered into conscious awareness through feedback, discovery, or reflection. For example, Maria emphasised her lack of conscious awareness of eye-contact in the following statement ‘for my personal case, we don’t realise that we’re not making eye-contact directly.’ Maria’s pronoun shift from the first person singular to plural, indicates an internal shift from focusing on oneself, towards a concern for autistic people as a collective group, suggesting that a lack of conscious awareness of eye-contact is understood by Maria to be a shared phenomenon. For Maria, atypical eye-contact is not something that she is typically cognisant of during social interactions, but it is brought into her awareness through her father’s frequent feedback:

‘I might not be looking directly in their eyes. But I don’t realise that. I think, if I’m looking at the person, I’m kind of looking up and down, or it’s hard, so I think my dad will always notice it.’ (Maria).

Maria’s ambivalent feelings towards feedback regarding her atypical eye contact was evident in her statement ‘I’m quite glad he said it, but it is sometimes a bit hard to hear.’

Conversely, Shane and Lauren described their increased awareness of eye-contact following their ASD diagnosis: ‘she just took out the form of traits, and what not, of Asperger’s and I thought ‘That sounds like that makes sense’ [laugh]. And I guess that it wasn’t until that that I thought ‘Hold on, eye-contact!’’ (Shane).

In his utterance, Shane exclaims his surprise in his words ‘hold on, eye-contact!’, suggesting that past experiences of eye-contact entered conscious awareness, and made ‘sense’ under the diagnosis of ASD. An increased awareness of eye-contact led to different internalised beliefs and emotional reactions. For example, following her diagnosis, Lauren felt that she would have to ‘make sure that [she] was making some eye-contact’. Her assertion that she would have to ‘make sure’ indicates her belief that eye-contact is necessary in social interactions, while her use of the quantifier ‘some’ suggests that, despite its importance, eye-contact is experienced as an effortful task. Similarly, John experienced ambivalent emotions about his increased awareness of eye-contact after it was ‘pointed out to him’, as it provided him with both a framework of understanding, and also an internal pressure to conform; ‘In some ways it was a revelation, and in some ways it was inconvenient [laugh].’ (John).

On the other hand, Jason ‘eliminated dairy’ from his diet and became cognisant that he had not been making eye-contact up to that point. Jason understood the ‘casein in dairy’ as influencing his autistic traits, particularly his eye-contact, and described how he ‘started to look people in the eyes’ following his dietary changes. Jason’s developmental understanding of eye-contact, and his sense of excitement about his new initiation of eye-contact was evident in his use of simile, as he described this experience as akin to a ‘child waking up to some sort of experience.’

#### Subtheme 2: Motivation to Make Eye-Contact in the Context of Autistic Identity

Participants’ motivations to make eye-contact appeared to be influenced by their perceptions of both autism and societal expectations regarding social communication. Participants spoke about intrinsic and extrinsic motivating factors. For example, for both Ryan and Jason, eye-contact was understood as a means of ‘connecting’ with others, in the context of their social difficulties associated with autism. Using exercise as a simile, Ryan highlights the absence of his natural motivation to establish eye-contact, while also identifying the social reward that serves as a positive reinforcement: ‘It’s kind of like doing exercise, you never really want to do it but when you actually do it you feel better after doing it.’ (Ryan).

While Jason also experiences a sense of connection with others, he described an inner conflict between his desire to make eye-contact and his persistent struggle to maintain eye-contact on a multisensory level: “If I look at people then I’m connecting more and then another epiphany of like ‘I can’t look at people and talk to them meaningfully at the same time.’” (Jason). Other participants were motivated to make eye-contact by a desire to ‘fit in’ and seem ‘normal’. This appeared to be influenced by their perception of ASD as a diverse spectrum of ability, in which autistic traits may differ according to where people exist on the spectrum. For example, Maria feels that her autism is not ‘very bad’, and does not want being autistic to define her. She therefore attempts to camouflage her autistic traits in order to make friends. Similarly, Jack described his social skills as stronger in comparison to other autistic people he knows. His assertion that eye-contact is a ‘required skill as a human’ depicts his belief that eye-contact is expected from others during social interactions.

Conversely, three participants do not report intrinsic motivation to make eye-contact, but rather engage in compensatory behaviours for extrinsic motivation, such as avoiding negative social outcomes. For example, Shane ‘never saw much use’ for eye-contact, and expressed his belief that it was unnecessary for communication, as he doesn’t ‘need to’ make eye-contact with his autistic friends. Similarly, John perceives eye-contact to be for the ‘benefit of neurotypicals’, and highlighted that bi-directional differences in autistic and non-autistic behaviours can influence interpretations of reduced eye-contact, as it ‘doesn’t necessarily mean anything’ if autistic people are not making eye-contact.

### GET 2: Phenomenology of Eye-Contact

This GET describes participants’ phenomenological experience of making eye-contact, including their processing difficulties and the experience of eye-contact with familiar and unfamiliar others.

#### Subtheme 1: ‘Eye-Contact is Part of Thinking’: Processing Difficulties

One of the most striking aspects of participants’ accounts was their description of processing while trying to maintain eye-contact. All participants spoke about difficulties related to cognitive and social processing during social interaction. For example, Jason conceptualises social interaction as a ‘multifaceted task’, which echoes John’s experience of ‘decision work’. In general, participants referred to the challenge of ‘multi-tasking’ during conversation and difficulties processing multi-sensory components simultaneously: ‘I might look at someone in the eyes for a few seconds but be able to say absolutely nothing, but then when I look away I can say it.’ (Shane)

‘being so focused on the other person takes a lot of effort. It’s kind of- to just stay in conversation and not drift off into your own thoughts. Particularly when you are in conversation; you kind of think of something you want to say- it’s trying to focus on the person, and what they’re saying, and maintain dialogue and be present, I think is something hard.’ (Maria).

In these quotes, both Shane and Maria’s description of competing cognitive demands during a social interaction implies that conscious eye-contact depletes their cognitive capacity to attend to other aspects of the conversation, such as thinking about what to say and focusing on the speech acts of their conversation partner. In her statement, Maria highlights the effort of not ‘drifting off’ into her thoughts, which indicates her natural tendency to focus on her own consciousness during social interactions. Ryan echoes this point, as he described eye-contact often feeling ‘unnecessary’ given he has ‘thoughts in [his] mind that [he] like[s] to think about’. Shane’s tendency to look away implies that averting his gaze allows him to focus on his own thought process and preserve attentional and cognitive resources to maintain a social interaction. Indeed, many participants described averting their gaze in order to feel ‘embodied’ (Jason), ‘tap into feelings and thoughts’ (Jason), ‘think of what to say’ (John), and ‘process what to do next’ (Jack).

For some, eye-contact was also related to difficulties managing sensory input, as expressed by Shane ‘looking someone in the eyes while speaking to them or listening to them cuts off my mental imagery as well as my ability to take in their words.’ In his statement, Shane understands eye-contact as impeding his ability to engage meaningfully in conversation, as it ‘cuts off’ his ability to speak and listen to others.

All participants spoke about attempts to process social information during social interactions. This appeared to be a conscious attention to social information, which contrasts to the neurotypical experience in which this social information would be absorbed effortlessly and without conscious thought. For example, some participants looked towards the face in an attempt to gauge information about how they are performing socially, or make a social decision: ‘You will look at that person to sort of, how much should I say? I might look at them and try and gauge that.’ (Maria). ‘You’re trying to get a hint of “are they interested”, or maybe facial expressions.’ (Jack).

However, despite their efforts, some reported difficulties reading social information, such as body language (Kevin, John) facial expressions (Shane, Lauren, John), and inferring others’ mental states (Kevin, John, Lauren), as expressed by John: ‘But when I’m looking at somebody’s face though, I’m thinking, I’m sort of thinking that a normal person could read a whole load of things by doing this, but I can’t [laugh].’ (John). Here, John demonstrates a self-reflexive process in which he considers his experience of eye-contact in relation to a ‘normal person’, implying some conscious awareness that he is missing out on information accessible to neurotypicals.

In addition, three participants spoke about difficulties maintaining eye-contact in group situations given the increased cognitive demands of trying to manage interactions with multiple people, as highlighted by Kevin who feels that he ‘gets lost’ and ‘can’t keep [eye-contact] going.’

#### Subtheme 2: Familiar Others Mediating Affective Arousal and Eye-Contact

Many participants found that their experience of making eye-contact was dependent on ‘how relaxed or stressed’ they are (John). In particular, affective arousal levels appeared to be influenced by their familiarity with their conversation partner: ‘I find…the more you know a person the easier it is to do eye-contact with them.’ (Ryan). Participants distinguished between their experience of making eye-contact with familiar and unfamiliar others. For most participants, eye-contact with familiar others was perceived to be easier due to several factors, including predictable emotional reactions (John, Maria), familiar others ‘get’ them (Maria; Sam), familiar others have no ‘expectations’ regarding eye-contact (Shane, John), and some participants identified the importance of close relationships (Shane, Jack), and having ‘nothing to hide’ (Kevin) with familiar others. The conscious process of making eye-contact was experienced as being ‘hard’ (Maria), and a lot of ‘work’ (John). As such, John’s sense making of eye-contact with familiar others is that he can ‘turn the conscious bit off’ and feel more ‘relaxed’, which denotes that reduced cognitive effort and decreased affective arousal positively influence his felt experience of eye-contact.

On the other hand, eye-contact with unfamiliar others increased levels of affective arousal in the context of being unable to read ‘ambiguous’ behaviour (John, Kevin), felt pressure to perform socially (Lauren, Kevin), and concerns of ‘giving off a bad impression’ (Maria). This experience of increased self-consciousness was echoed by Lauren and Jason who felt ‘hyper-aware’ when interacting with unfamiliar others, as illustrated in the following quote: ‘I’d be looking at them, and then when they look at me it’s like a reflex to just look away. Like I wouldn’t continue looking at them.’ (Lauren).

Lauren’s description of her automatic reaction to ‘look away’ and her report that she is ‘worried what they would think’ of her, implies that she equates direct eye-contact with increased concern about how she is perceived by others. For Jason, his felt experience of eye-contact is ‘exponentially less laborious’ following reductions in his ‘social anxiety’, implying a possible interactional process where pre-existing social anxiety makes eye contact more difficult to tolerate and the eye contact itself adding to the experience of anxiety.

### GET 3: Strategies to Make Eye-Contact

Participants reported a knowledge of the social function of eye-contact, such as conversational turn-taking, signalling an intention to communicate, and inferring how others are thinking and feeling. Thus, strategies were developed to try and cope in a social world.

Eye-contact required conscious thought and effort, and some participants described their attempts to manage appropriate eye-contact so that they were not ‘staring’, as highlighted by Maria: ‘You just sort of look away because you don’t want to be seen as staring.’

By referring to herself in the second person, it is possible that Maria is creating an emotional distance from the content of her statement, in an attempt to relieve some of the shame that she experiences as a result of failing to meet the standards she imagines are expected by neurotypicals. Other participants employed compensatory behaviours in order to pass as ‘normal’ or for the benefit their conversation partner; for example, ‘practicing’ (Ryan), ‘looking away occasionally to avoid staring’ (John), looking in the ‘general direction’ of the eyes (Shane), and sitting at a ‘perpendicular angle’ to avoid direct eye-contact (Lauren). Shane described strategic use of eye-contact:

‘I say in my core sentence whatever I have to say and then for a brief second, I slightly look them in the eye or I totally do. And that’s not comfortable either but at least I’ve said what I needed to say.’ (Shane).

This excerpt illustrates the effortful compensatory behaviours that some autistic people engage in to maintain eye contact, and it is evident in Shane’s experience that discomfort is felt while trying to behave in line with societal expectations regarding eye-contact.

Conversely, both Ryan and Jason experienced adaptation following conscious attempts to establish eye-contact:

‘Before when I tried to make eye-contact I could do it almost too hard in a sense. But I can do it more, I can do it better now like. At first I was being kind of surgical with practicing it. Now it comes a bit more naturally to me.’ (Ryan).

Both Ryan and Jason reported increased frequency of eye-contact over time, and described their awareness of ‘eye colours’ when establishing a direct gaze, which could suggest an intense fixation on the eyeball itself, or possibly just more acute observational awareness. Thus, while the frequency and their felt experience of eye-contact has changed over time, their descriptions suggest an atypical quality to their attempts at eye-contact.

While many described their attempts to make eye-contact, John believes that autistic people should have a choice to attempt eye-contact, or receive intervention: ‘As long as it means having the option, I think [it] is good, right? I think it should be a choice, rather than something mandatory or drummed into people.’ (John).

John’s use of the phrase ‘drummed into people’ highlights that in the past, autistic people may not have been given choice about interventions and expresses his wish for autistic people to initiate and control their own actions relating to eye-contact, which is expressed through his words ‘option’ and ‘choice’.

## Discussion

The aim of this study was to explore autistic people’s experiences of eye-contact, using an IPA qualitative methodology. The analysis revealed three interrelated GETs: Awareness and motivation for eye-contact, Phenomenology of eye-contact, and Strategies to make eye-contact. The GETs are discussed below to consider how the current findings relate to, and expand upon existing knowledge.

Consistent with experimental eye-tracking research (Chita-Tegmark, 2016; Frazier et al., 2017), participants in this study experienced atypical eye-contact, characterised by unusual eye-contact patterns, and reduced attention to the eyes. Participants experienced various levels of conscious awareness of eye-contact, and most participants became aware of their differences in eye-contact over time, either through feedback, prior to diagnosis, or through the process of increased self-awareness, suggesting that avoidance of direct gaze was not initially occurring on a conscious level. This finding is consistent with eye-tracking research, in which an absence of preferential unconscious processing of direct gaze has been observed (Akechi et al., 2014; Madipakkam et al., 2017). However, it is important to note the inherent limitations of comparing experimental results with phenomenological accounts. The emotional valence associated with increased awareness of eye-contact is important, as it provided some participants with a framework for understanding and a sense of determination to develop this skill, while others felt a heightened pressure to conform, indicating their awareness of societal expectations regarding mutual eye-contact. Participants reported that the experience of cognitive load while engaging in eye contact during social interactions was particularly challenging and this concords with some other studies in this area (Kliemann et al., 2012; Tanaka & Sung, 2016).

The findings from this IPA study also contribute to existing qualitative research exploring the way in which autistic people experience and manage social interactions. For example, a recent study exploring autistic participant’s experience of speech perception identified contributing factors to speech perception difficulties, including acoustic and non-acoustic factors such as multi-sensory processing and social cognition (Sturrock et al., 2022). They also reported that participants identified deliberate and reflexive coping mechanisms that were employed, such as self-awareness and self-advocacy, communication tactics, and developing auditory skills (Sturrock et al., 2022). Furthermore, another qualitative study identified camouflaging behaviours used by autistic adults in everyday social interactions, including masking, superficial social engagement, communicating in line with non-autistic norms, and active self-presentation (Cook et al., 2022). Thus, the current study contributes to the growing body of qualitative research exploring autistic people’s first-hand experience of social interaction.

In this study, participants reported motivations to make eye-contact that appeared to be largely influenced both by their perceptions of autism and a social expectation from the general population. While most participants held an understanding of non-autistic peoples’ value of eye-contact, some felt that eye-contact was unnecessary in order to communicate with other autistic people. This is consistent with previous qualitative literature highlighting that autistic peoples’ interactions with each other are fundamentally different to interactions with non-autistic others (Crompton et al., 2020). However, despite these beliefs and consistent with previous research, many participants reported camouflaging their atypical eye-contact, by masking, or adopting compensatory strategies. The tendency for autistic people to modify their social behaviours and autistic traits in order to adapt or cope within a social world has been found in other studies (Cook et al., 2022; Hull et al., 2017). In the current study, some participants were internally driven to make eye-contact in order to establish specific goals, such as friendships, or to appear ‘normal’, particularly those participants who perceived autism as a spectrum of varying ability. However, others engaged in camouflaging behaviours due to their perceptions of how one should behave in society, despite their own beliefs about eye-contact. This is important as camouflaging comes with risks – some autistic people develop strategies built on their innate strengths to appear more socially competent, however, hiding underlying difficulties can reduce access to support services (Tierney et al., 2016).

Consistent with previous qualitative findings, some participants reported an awareness of ‘staring’ during social interactions (Trevisan et al., 2017). Participants employed conscious masking strategies, such as looking away, in order to avoid engaging in this behaviour. Several participants also engaged in compensatory strategies to appear neurotypical in certain contexts. However, there is ample evidence to suggest that autistic participants who engage in compensatory behaviours experience this as emotionally and cognitively demanding, involving considerable conscious effort and adaptability (Livingston et al., 2020). Indeed, many participants reported the psychological and emotional impact of employing compensatory strategies, including on the ability to process information, and some described the process of making direct eye-contact as effortful. Conversely, some participants described remediation of their difficulties with eye-contact, in which they experienced more frequent and comfortable eye-contact. However, their descriptions of mutual eye gaze suggest an unusual quality to their eye-contact may have persisted. It is plausible that with repeated efforts to establish direct eye-contact, these participants experienced a reduction in physiological arousal through habituation, and increased social rewards over time. Therefore, this improvement in the participants’ felt experience of eye-contact may not reflect alterations or alleviations of underlying cognitive differences, but rather, the participants’ enhanced experience of making eye-contact (Livingston et al., 2019).

During conversation, the participants used eye-contact to monitor the other person’s availability, interest, reactions and emotions. However, participants experienced specific cognitive activity that influenced their phenomenological experience of eye-contact during social interactions. Consistent with other qualitative reports (Trevisan et al., 2017), participants described difficulties multi-tasking, and processing information from multiple sensory modalities at one time, particularly maintaining eye-contact while listening or speaking to others. Indeed, multisensory integration difficulties, particularly pertaining to audio-visual integration, have been identified in ASD (Feldman et al., 2018). In the current study, participants reported their tendency to ‘look away’ in order to process information or think about what they will say. This is consistent with experimental research in which both autistic and non-autistic participants showed increased attention to their conversation partner’s eyes when listening, rather than speaking, suggesting that autistic and non-autistic participants could modulate their social attention depending on conversational phase (Freeth & Bugembe, 2019). Averted gaze during conversation may be explained by the ‘cognitive load hypothesis’, in which gaze aversion is understood as a cognitive control strategy that is used by both typical and atypical populations in complex interactions (Doherty-Sneddon et al., 2013). Therefore, in the current study, participants’ tendency to avert their gaze during conversation may serve a cognitive and social function, as they can focus on their internal processing, and reduce the cognitive load of attending to each aspect of the social interaction.

In addition to more general cognitive processing, social processing differences have also been identified as a reliable feature of ASD (Frazier et al., 2017). Indeed, some participants experienced difficulties mentalising and gauging social information from the face, suggesting that social processing difficulties may influence their experience of eye-contact. This was experienced as distressing and frustrating by most participants and echoed findings from previous qualitative work, in which autistic participants understood that social information could be obtained from the eyes, but they personally could not detect it (Trevisan et al., 2017). This finding is consistent with the mindblindness model (Baron-Cohen, 1994), which postulates that difficulty with inferring the mental states of others is associated with atypical eye-contact.

Participants also described a distinct phenomenological difference when making eye-contact with familiar and unfamiliar others. Some participants felt it was more comfortable to make eye-contact with familiar others, as they could predict their emotional responses, as opposed to the ambiguous behaviours of unfamiliar others. Thus, eye-contact with unfamiliar others was experienced as more cognitively effortful, but this effort appeared to be mitigated when interacting with a familiar social partner. Previous research has found that emotional processing difficulties observed in ASD may be mediated by familiarity (Nuske et al., 2014). Therefore differences in participants’ felt experience of making eye-contact with familiar and unfamiliar others may be a result of more frequent emotional learning opportunities that comes from familiar others, that may not generalise to unfamiliar people.

Heightened self-consciousness and increased awareness of autistic traits was another factor influencing the felt experience of eye-contact with unfamiliar others. Self-consciousness requires an understanding of the self in relation to external standards, as well as an appraisal of how one’s behaviour is evaluated by others (Davidson et al., 2017). Recent qualitative research found that autistic participants are more aware of their minority status when interacting with non-autistic peers (Crompton et al., 2020), and autistic participants have been found to resemble non-autistic participants in their susceptibility to social desirability and self-enhancement pressures (Gernsbacher et al., 2020). Therefore, while some participants described increased physiological activity in response to direct eye-contact, it is plausible that interacting with unfamiliar others induces feelings of shame, and anxiety, which ultimately influences levels of affective arousal in response to direct gaze.

### Limitations and Directions for Future Research

Several limitations should be considered in the interpretation of this study’s results. Firstly, as with any IPA study, the focus is on the in-depth examination of the individual experience of a small sample of autistic participants, without the aim of being representative or generalizable. This study only included participants with no co-occurring intellectual disability to ensure relative homogeneity of the sample as is required in IPA studies. Therefore, future research may consider using more flexible methodologies in order to consider the experiences of less cognitively able autistic individuals (Leedham et al., 2020).

In the context of COVID-19, the interviews were conducted online, which had implications for the researcher forming an impression of participants’ use of eye-contact during the interview. Although meaningful and rich data was obtained from participants using IPA methodology, challenges with language difficulties were noted, and some participants demonstrated limited capacity to introspect on their thoughts and feelings, and articulate their emotional or physiological experiences. Therefore, future research may consider interviewing both autistic participants and a close relative or partner, in order to integrate a first and third person perspective on eye-contact. Future research may also gather additional demographic information, including socioeconomic status and educational attainment level, in order to consider individual factors that may influence participants’ experience of eye-contact.

## Conclusion

The findings of this study reflect the heterogenous phenomenological experiences of eye-contact among a sample of autistic adults. Based on the findings, eye-contact is often a conscious process, and many employ camouflaging strategies to mask or compensate for their atypical eye-contact. However, despite their best efforts to make eye-contact, factors such as cognitive and social processing difficulties appear to inhibit effective eye-contact. This has implications for psychological intervention, as it suggests that other cognitive processes may need to be targeted concurrently. Additionally, it highlights the need for clinicians to consider what accommodations may be helpful for autistic adults and how they can communicate this need in social settings. Not making eye-contact may be a choice that is easier to facilitate in all-autistic settings, but many neurotypicals may not be aware of the degree to which eye contact can be uncomfortable for autistic people. This is consistent with some of the aims of the neurodiversity movement, which advocates for the development of a diversity-valuing society in which autistic people feel safe to express their autistic traits, and reduction in the pressure to conform (Cage & Troxell-Whitman, 2020).
